# Geometric accuracy of 3D coordinates of the Leksell stereotactic skull frame in 1.5 Tesla- and 3.0 Tesla-magnetic resonance imaging: a comparison of three different fixation screw materials

**DOI:** 10.1093/jrr/rru064

**Published:** 2014-07-17

**Authors:** Hisato Nakazawa, Yoshimasa Mori, Osamu Yamamuro, Masataka Komori, Yuta Shibamoto, Yukio Uchiyama, Takahiko Tsugawa, Masahiro Hagiwara

**Affiliations:** 1 Department of Radiological Sciences, Nagoya University Graduate School of Medicine, Nagoya, Aichi, Japan; 2 Nagoya Radiosurgery Center, Nagoya Kyoritsu Hospital, Nagoya, Aichi, Japan; 3 Department of Radiology, Nagoya City University Graduate School of Medical Sciences, Nagoya, Aichi, Japan; 4 East Nagoya Imaging Diagnosis Center, Nagoya, Aichi, Japan

**Keywords:** magnetic resonance imaging, computed tomography; stereotactic localization, stereotactic radio-surgery, image distortion

## Abstract

We assessed the geometric distortion of 1.5-Tesla (T) and 3.0-T magnetic resonance (MR) images with the Leksell skull frame system using three types of cranial quick fixation screws (QFSs) of different materials—aluminum, aluminum with tungsten tip, and titanium—for skull frame fixation. Two kinds of acrylic phantoms were placed on a Leksell skull frame using the three types of screws, and were scanned with computed tomography (CT), 1.5-T MR imaging and 3.0-T MR imaging. The 3D coordinates for both strengths of MR imaging were compared with those for CT. The deviations of the measured coordinates at selected points (*x* = 50, 100 and 150; *y* = 50, 100 and 150) were indicated on different axial planes (*z* = 50, 75, 100, 125 and 150). The errors of coordinates with QFSs of aluminum, tungsten-tipped aluminum, and titanium were <1.0, 1.0 and 2.0 mm in the entire treatable area, respectively, with 1.5 T. In the 3.0-T field, the errors with aluminum QFSs were <1.0 mm only around the center, while the errors with tungsten-tipped aluminum and titanium were >2.0 mm in most positions. The geometric accuracy of the Leksell skull frame system with 1.5-T MR imaging was high and valid for clinical use. However, the geometric errors with 3.0-T MR imaging were larger than those of 1.5-T MR imaging and were acceptable only with aluminum QFSs, and then only around the central region.

## INTRODUCTION

Magnetic resonance (MR) imaging with 1.5 Tesla (T) is usually used for Gamma Knife stereotactic radiosurgery (GKSRS) treatment planning (GK and GammaPlan treatment-planning device, Elekta, Tokyo) and planning of stereotactic surgery using a Leksell stereotactic skull frame. Recently, higher magnetic field 3.0-T MR imaging, which provides a better neuroanatomical image, has become increasingly popular for clinical diagnostic imaging, although its safety with the metal skull frame is not completely validated yet. Previously, our institute reported that the geometric uncertainty of 1.5-T MR imaging was minimal when tungsten-tipped aluminum quick fixation screws (QFSs) (Elekta, Tokyo) were used for skull frame fixation [1]. Since then, whole aluminum QFSs without a detachable tungsten tip, and titanium QFSs (constructed from a non-magnetic material causing less artifacts in X-ray computed tomography (CT)) have become commercially available. Tungsten-tipped aluminum QFSs are generally used in 1.5-T MR imaging for clinical GKSRS; however, in 3.0-T MR imaging their use is not recommended by a vender (ELEKTA) because of the possibility of skin injury [2]. Both aluminum and titanium QFSs have been developed for 3.0-T MR imaging. Although the aluminum QFSs are disposable, titanium QFSs are usually used for 3.0-T MR imaging. In a previous paper, however, the heating of tungsten-tipped aluminum and titanium QFSs during 3.0-T MR imaging was reported to be negligibly small [3].

The geometric accuracy of 3.0-T MR imaging using titanium QFSs has been investigated [4–7]. Distortion correction algorithms and optimized scan protocols were employed to achieve acceptable image distortion. However, there have been few publications on the geometric inaccuracy caused by different QFSs. The aim of this study was to evaluate the geometric image distortion produced by three types of cranial QFSs constructed of different materials, namely aluminum, aluminum with a tungsten tip, and titanium, for both 1.5-T and 3.0-T MR imaging.

## MATERIALS AND METHODS

### Evaluation of stereotactic coordinates

**Fig. 1.**
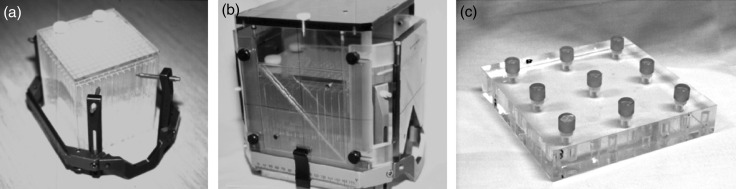
Phantom A (a cubic-shaped phantom including matrix structure) was fixed to a Leksell G skull frame (**a**). A Leksell stereotactic indicator box for MR imaging was mounted on the frame with Phantom A (**b**). Phantom B: an acrylic plate phantom with nine small cylinder-shaped baths (**c**).

Two types of phantoms were used for this study: a grid-pattern acrylic box phantom (Phantom A) (Fig. 1a and b) and an acrylic plate with nine cylinder-shaped baths (Phantom B) (Fig. 1c). Phantom A consists of a matrix-shaped acrylic structure inside a cubic box of 150 × 150 × 150 mm, which is approximately the same volume as the average human adult head. The spatial interval of the grid in Phantom A is ∼10 mm. Phantom B is embedded with a cylinder inside a cubic-shaped plate of 150 × 150 × 25 mm. Each cylinder of Phantom B is 8 mm in diameter and 10 mm in height. The cubic Phantom A and the baths of Phantom B were filled with a solution of a mixed gadolinium and iodinated contrast medium (Gd + I, 0.1 mmol/kg). The phantoms were mounted on the Leksell stereotactic frame (Elekta, Tokyo) using QFSs through single-use plastic insulator parts (Elekta, Tokyo) by four aluminum posts (anterior post height: 123 mm, posterior post height: 85 mm). Phantom B was firmly fixed on the superior plane of Phantom A during MR imaging. Stereotactic fiducial indicator boxes (Elekta, Tokyo) were attached on the Leksell frame to provide a stereotactic coordinate system for treatment planning. The phantoms were also scanned with a CT scanner by attaching the CT fiducial box with low artifact copper strings as a fiducial reference.

**Fig. 2.**
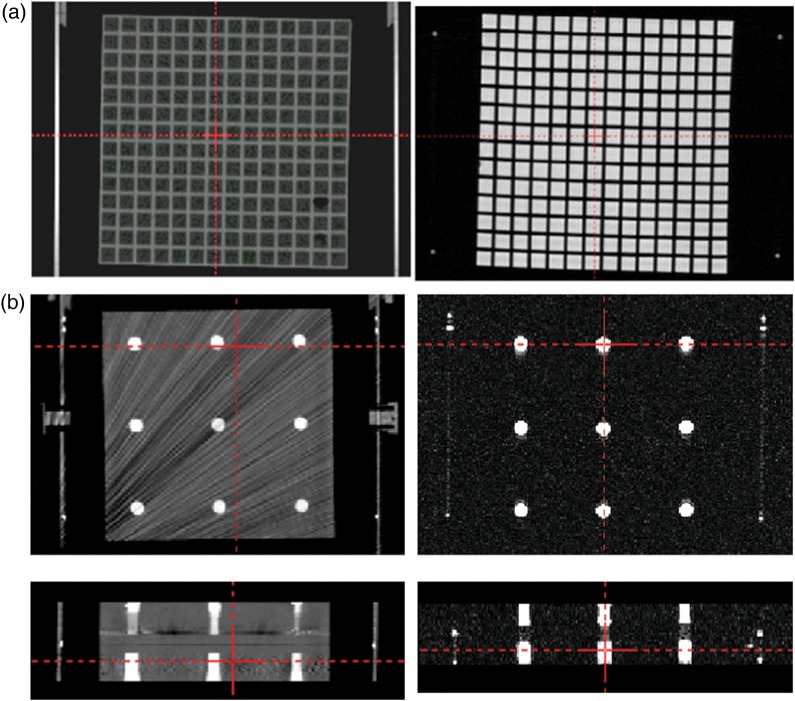
Axial images of Phantom A including matrix structure on CT (left) and MR imaging (right). The difference in coordinates for the same gridpoint between CT and MR images is measured by using the crossing point tool in a planning system (a). Axial images (upper) and coronal images (lower) of Phantom B with nine baths on CT (left) and MR imaging (right) (b).

A Signa HDxt 1.5-T MR imaging scanner (GE Healthcare, Tokyo), Signa HDxt 3.0-T MR imaging scanner (GE Healthcare, Tokyo) and TruePoint Biograph 40 CT scanner (Siemens, Tokyo) were used. Acquisition of MR images with both 1.5-T and 3.0-T MR imaging was performed with quadrature head coils (GE, transmit/receive, 28-cm diameter). Details of the acquisition parameters for 3D spoiled gradient recalled acquisition in the steady state (3D-SPGR) and CT scanning are shown in Table 1. The slice thickness was 1.0 mm with zero gap, and the pixel size of images was 1.0 mm (*x*) by 1.0 mm (*y*) for both 1.5-T and 3.0-T MR imaging. The pixel size of CT images was 0.5 mm (*x*) by 0.5 mm (*y*). The orientations of the axes in the stereotactic space coordinates system were *x* as left–right direction, *y* as anterior–posterior direction, and *z* as superior–inferior direction. All CT and MR images were exported to the Leksell GammaPlan (LGP) (Elekta, Tokyo) treatment-planning software workstation. These images loaded in LGP were registered with the stereotactic coordinate system using the fiducial markers on the localizer box. To assess the correlation between MR imaging and CT, the coordinates of crossing points of the matrix structure in Phantom A (around *z* = 50, 75, 100, 125 and 150) were measured to investigate the *x*- and *y*-dimensions (around *x* = 50, 100 and 150, and *y* = 50, 100 and 150) on different axial planes (*z* = 50, 75, 100, 125 and 150) (Fig. 2a). To examine the *z*-dimension, the coordinates of the center of each of the cylinder-shaped baths of Phantom B were measured (around *z* = 50, 75, 100, 125 and 150, *x* = 50, 100 and 150, and *y* = 50, 100 and 150) (Fig. 2b). Three types of QFSs were used. Measurement of the coordinates was manually performed three times for each of the measuring points on the LGP monitor.

**Table 1.** Scanning parameters for 3D-SPGR with 1.5-Tesla MR imaging, 3.0-Tesla MR imaging and CT as a reference for stereotactic coordinates

	3D-SPGR			CT
	1.5-Tesla MR imaging	3.0-Tesla MR imaging		
Plane	Axial	Axial	Scan mode	Non-helical
Mode	3D	3D	Voltage (kV)	120
TE (ms)	Min full (5.06)	Min full (3.2)	Current (mA)	170
ETL	1	1	Number of slices	120
Flip angle (degree)	30	30	Slice thickness	1.0
Bandwidth (kHz)	15.63	15.63	Slice gap (mm)	0
NEX	1	1	FOV (cm)	30
FOV (cm)	24	24		
Matrix size	256 × 256	256 × 256		
Slice thickness (mm)	1.0	1.0		
No. of slices	190	190		
Scan time (min)	8:26	8:47		

TE = echo time, TR = repetition time, ETL = echo train length, NEX = number of excitations, FOV = field of view, SPGR = spoiled gradient recalled acquisition in the steady state.

### QFS-induced MR image distortion

**Fig. 3.**
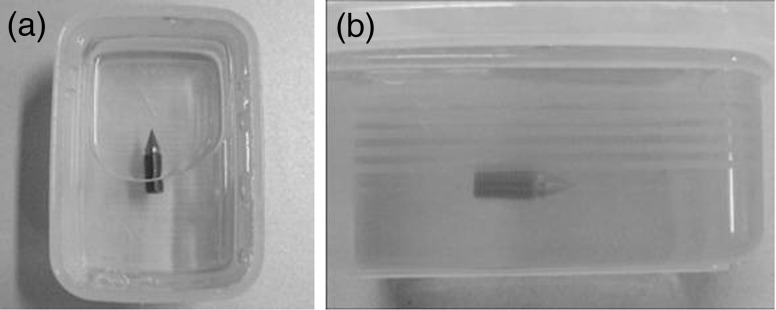
The quick fixation screw (QFS) was embedded in gelatin and its image was acquired using both 1.5-T and 3.0-T MR imaging scanners in order to visualize levels of susceptibility artifacts around the QFS. View from above (a) and lateral view (b).

MR images of the three types of QFSs embedded in gelatin (Fig. 3) were taken by 3D-SPGR with the same pulse sequence (using both 1.5-T and 3.0-T scanners) to assess the distortion of the images.

## RESULTS

**Fig. 4.**
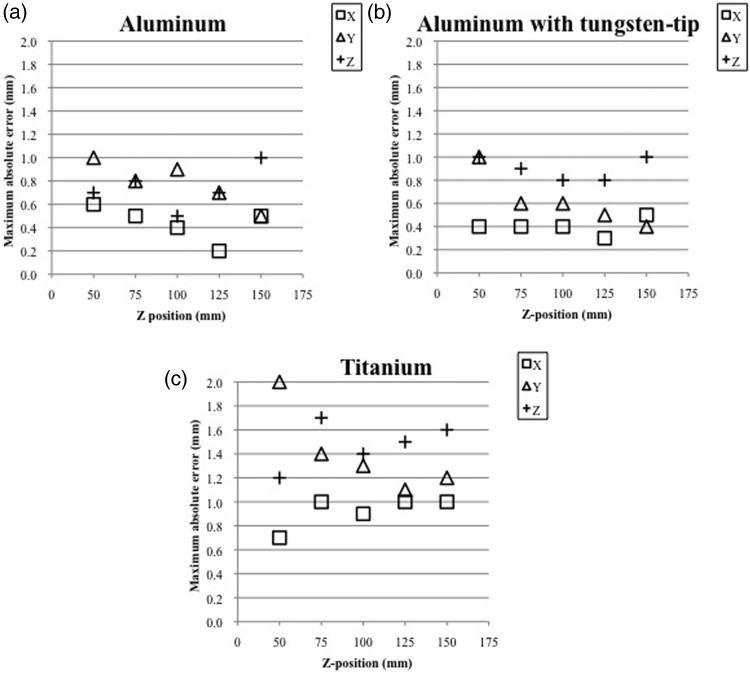
Maximum absolute error (mm) of coordinates among nine target points in each dimension on each z-plane, compared with 1.5-T MR images and CT images for three types of cranial screws of different materials: aluminum (a), aluminum with a tungsten tip (b), and titanium (c). The x- and y-coordinates were measured for Phantom A, and the z-coordinate was measured for Phantom B.

**Fig. 5.**
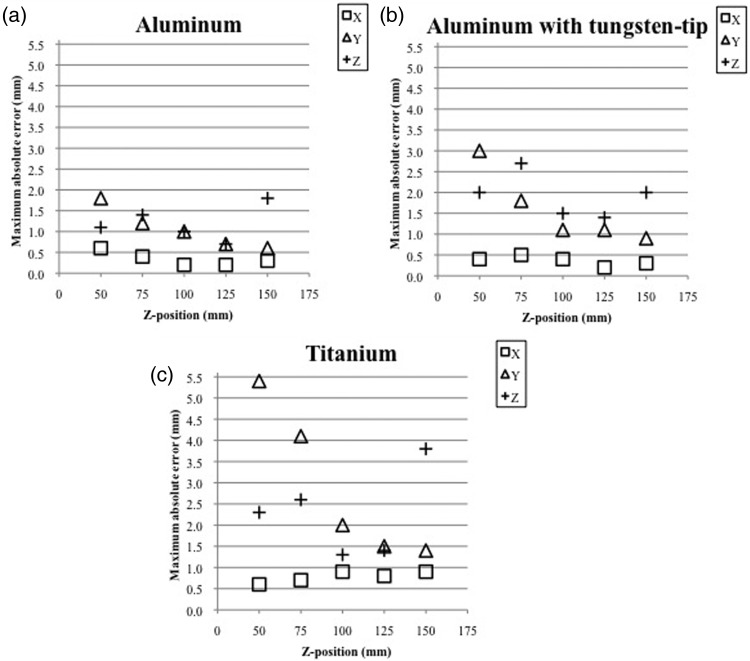
Maximum absolute error (mm) of coordinates among nine target points in each dimension on each *z*-plane, compared with 3.0-T MR images and CT images for three types of cranial screws of different materials: aluminum (**a**), aluminum with a tungsten tip (**b**), and titanium (**c**). The *x*- and *y*-coordinates were measured for Phantom A, and the *z*-coordinate was measured for Phantom B.

Figures 4 and 5 show the maximum absolute errors in the coordinates for three different QFSs on 1.5-T MRI images and 3.0-T MR images, determined by comparing the images with the CT images, respectively. Tables 2 and 3 show the average ± 1 standard deviation of the deviations of the coordinates for the QFSs constructed of the three different materials on the 1.5-T MR images (Table 2) and the 3.0-T MR images (Table 3) by comparing them with those on the CT images in each *z*-plane. For both 1.5-T and 3.0-T MR imaging, the deviations of the coordinates showed minimum values around the center, (*x*, *y*, *z*) = (100, 100, 100), with all three types of QFS. With aluminum and tungsten-tipped aluminum QFSs, the errors of the coordinates were <1.0 mm on the 1.5-T-MR images. With titanium QFSs, the errors were between 1.0 and 2.0 mm on the 1.5-T MR images. Using 3.0-T MR imaging, the errors with QFSs of tungsten-tipped aluminum and aluminum were <1.5 and <1.0 mm, respectively, around the center and up to 3.0 mm around the vertex area. The errors with titanium QFSs were between 1.0 and 2.0 mm around the center and up to 5.4 mm around the vertex area. With aluminum screws, the geometric deviations were the smallest and were below the size of the image matrix except at peripheral locations and near the frame base on both 1.5-T and 3.0-T MR imaging.

**Table 2.** Mean ± 1 standard deviation (mm) for deviations of coordinates among nine target points in each dimension on each z-plane, compared with 1.5-Tesla MR imaging and CT for three types of cranial screws of different materials: aluminum (a), aluminum with tungsten tip (b), and titanium (c)

Table 2a			
z-position	x	y	z
50	0.4 ± 0.2	0.8 ± 0.2	0.5 ± 0.2
75	0.3 ± 0.2	0.7 ± 0.1	0.6 ± 0.2
100	0.2 ± 0.1	0.7 ± 0.2	0.3 ± 0.1
125	0.1 ± 0.1	0.5 ± 0.2	0.5 ± 0.2
150	0.3 ± 0.2	0.3 ± 0.2	0.7 ± 0.2
Table 2b			
*z*-position	*x*	*y*	*z*
50	0.2 ± 0.2	0.8 ± 0.2	0.7 ± 0.2
75	0.2 ± 0.2	0.6 ± 0.2	0.6 ± 0.3
100	0.3 ± 0.1	0.4 ± 0.2	0.6 ± 0.2
125	0.2 ± 0.1	0.3 ± 0.2	0.6 ± 0.2
150	0.3 ± 0.2	0.2 ± 0.2	0.8 ± 0.2
Table 2c			
*z*-position	*x*	*y*	*z*
50	0.6 ± 0.1	1.6 ± 0.4	1.0 ± 0.2
75	0.6 ± 0.2	1.1 ± 0.2	1.1 ± 0.4
100	0.5 ± 0.3	0.8 ± 0.3	0.9 ± 0.4
125	0.6 ± 0.2	0.6 ± 0.3	1.1 ± 0.4
150	0.8 ± 0.2	0.7 ± 0.4	1.2 ± 0.4

The *x*- and *y*-coordinates were measured for Phantom A and the *z*-coordinate was measured for Phantom B.

**Table 3.** Mean ± 1 standard deviation (mm) for deviations of coordinates among nine target points in each dimension on each z-plane, compared with 3.0-Tesla MR imaging and CT for three types of cranial screws of different materials: aluminum (a), aluminum with tungsten tip (b), and titanium (c)

Table 3a			
z-position	x	y	z
50	0.2 ± 0.2	1.5 ± 0.3	0.6 ± 0.4
75	0.2 ± 0.2	1.0 ± 0.2	1.0 ± 0.4
100	0.1 ± 0.1	0.8 ± 0.2	0.6 ± 0.4
125	0.1 ± 0.1	0.5 ± 0.2	0.5 ± 0.2
150	0.2 ± 0.1	0.4 ± 0.2	1.4 ± 0.4
Table 3b			
z-position	x	y	z
50	0.2 ± 0.2	2.5 ± 0.4	1.1 ± 0.6
75	0.2 ± 0.1	1.4 ± 0.4	1.6 ± 0.9
100	0.2 ± 0.1	0.6 ± 0.4	1.0 ± 0.5
125	0.1 ± 0.1	0.6 ± 0.3	0.8 ± 0.4
150	0.1 ± 0.1	0.5 ± 0.2	1.2 ± 0.8
Table 3c			
z-position	x	y	z
50	0.4 ± 0.2	4.7 ± 0.6	1.9 ± 0.4
75	0.5 ± 0.2	3.5 ± 0.5	2.0 ± 0.4
100	0.5 ± 0.3	1.4 ± 0.5	0.8 ± 0.4
125	0.6 ± 0.2	1.0 ± 0.4	1.0 ± 0.3
150	0.7 ± 0.2	1.1 ± 0.3	3.2 ± 0.5

The *x*- and *y*-coordinates were measured for Phantom A, and the *z*-coordinate was measured for Phantom B.

**Fig. 6.**
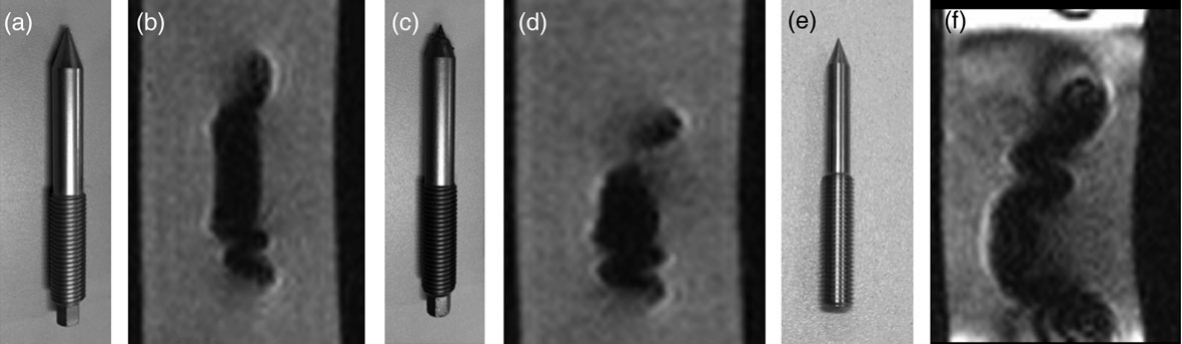
Aluminum quick fixation screw (QFS) (**a**), aluminum QFS with a tungsten tip (**c**), and titanium QFS (**e**), used during skull frame placement. The 3.0-Tesla MR images of each QFS embedded in gelatin (**b**, **d**, **f**). Distortion of the titanium QFS was remarkable. The tungsten-tipped QFS caused more distortion than the whole aluminum QFS.

Figure 6 demonstrates the image distortion by susceptibility artifacts around the three types of QFSs embedded in gelatin on both 1.5-T and 3.0-T MR imaging. The titanium QFSs caused the greatest signal defect and distortion among the three types of QFS, which was consistent with the results for the coordinate errors obtained with Phantoms A and B, as shown above.

## DISCUSSION

We evaluated the spatial accuracy of the coordinates system of the Leksell stereotactic skull frame using three different kinds of QFSs with both 1.5-T MR images and 3.0-T MR images. Prior to this study, the specific image distortions for both 1.5-T and 3.0-T MR imaging units were evaluated with a dedicated phantom (Japanese industrial standards phantom, 95–1108z type, Nikko Fines, Tokyo) used as a routine quality assurance tool. The distortion was l<0.2 mm/0.1% in both units, and there were no differences between the two units. Therefore, it seems that the difference in image distortion obtained in this study reflects the effects derived from the three types of QFS. For both MRIs, and with all three types of QFS, the errors of the coordinates were minimal around the center, (*x*, *y*, *z*) = (100, 100, 100) and maximal around the periphery. We speculate that the reason for this degradation of accuracy in the peripheral region is that quadrature head coils degrade in geometric image accuracy in the peripheral area in the superior–inferior direction. Under the 1.5-T magnetic field, the distortions caused by aluminum and aluminum with tungsten-tipped QFSs were similar and within the image matrix size over the entire measurement area accessible in GKSRS. The distortion caused by titanium QFSs was up to 2.0 mm, twice the image matrix size. On the other hand, with 3.0-T MR imaging, stereotactic localization errors were <1.0 mm only around the center of the fiducial indicator box when using aluminum QFSs. The errors using tungsten-tipped aluminum or titanium QFSs were >2.0 mm in most positions. We speculate that the difference in type of QFS influenced the accuracy of the stereotactic coordinate definition obtained using the fiducial in the localizer box.

In our study, titanium QFSs caused the largest distortion among the three types, although all errors were within the acceptable range on 1.5-T MR images. There have been a few reports describing satisfactory evidence of positional accuracy when using titanium QFSs in both phantom and clinical case studies with 3.0-T MR imaging [4–7]. Titanium has the advantage of causing fewer artifacts in CT. In our study, however, titanium also caused the largest distortion among the three types of QFSs on 3.0-T MR imaging and was not clinically acceptable. High-magnetic-field MR imaging is used to show detailed anatomic structures related to targets adjacent to important normal structures, such as the pituitary adenoma, craniopharyngioma, skull base meningioma, and trigeminal neuralgia. In order to use 3.0-T MR imaging for stereotactic dose planning of GK and planning of stereotactic surgery, further efforts should be made to reduce image distortion. In addition, spatial accuracy over the entire brain is necessary when multiple metastatic brain tumors are being treated.

Regarding image distortion, various factors can cause uncertainty in MR imaging, including instability of the main magnetic field, non-linearity of the gradient magnetic field, and susceptibility effects due to metallic materials (such as the localization frame and skull fixation screws) [7]. The distortion caused by gradient magnetic field non-linearity is usually small around the center of the magnetic field and increases with distance from the center. Watanabe *et al*. [4] have suggested that MR imaging should be taken near the central region of a head coil to minimize errors. They also compared internal marker positions on 3.0-T MR images with those on CT images using an original phantom, and the maximum *z*-coordinate errors were observed near the frame, which was consistent with our results.

Various methods for improving geometric accuracy have recently been suggested. They include distortion correction algorithms [4, 8–11], optimization of imaging protocols [7], and design of an original head coil and head immobilization device [12] for a certain high-magnetic-field-strength MR imaging system. First, the mathematical algorithm is an elegant method when using a homogeneous phantom filled with water, which only corrects for inhomogeneity in non-linearity of fields of view in the gradient magnetic field, but does not account for susceptibility effects caused by material heterogeneity including air, bone, and soft tissue [11]. Clinically, we need correction of unexpected positional deviations resulting from geometric distortion caused by complex structures in the human head, stereotactic skull frame and head screws. Second, concerning the acquisition parameters, in our study, the 3D-SPGR scanning parameters employed for 3.0-T magnetic fields were similar to the optimized parameters for GKSRS planning at 1.5 T. These parameter values were almost consistent with a 3.0-T MR imaging scan protocol reported by Zhang *et al*. [7]. On MR imaging for GKSRS, the scanning sequence is not limited to 3D-SPGR. Similar studies using other scanning sequences have recently been published [13–17]. Third, Jursinic *et al*. [12] developed an original MR-imaging head coil and immobilization device for better patient comfort and improvement of image quality while avoiding distortion. Artifacts due to patient movement during scanning were reduced, although the geometric accuracy was not improved.

Clinically, we only use 1.5-T MR images as a stereotactic reference image with a skull frame for GK treatment planning. Usually, 3.0-T MR images are only used as an additional image coregistered on the stereotactic reference image, after confirmation of accuracy of the coregistration function in the recent version of LGP [18].

## CONCLUSIONS

The geometric accuracy of the Leksell skull frame system with 1.5-T MR imaging was high and valid for clinical use with all three types of QFSs. However, the geometric errors with 3.0-T MR imaging were larger than those with 1.5-T MR imaging and were acceptable only around the center of the stereotactic coordinates when we used aluminum QFSs for skull frame placement.
